# The impact of digitalisation on the development of physical activity of older people (through the example of European Union countries)

**DOI:** 10.3389/fpubh.2026.1965028

**Published:** 2026-10-12

**Authors:** Zhaxat Kenzhin, Tursynzada Kuangaliyeva, Assem Aitkaliyeva, Yerbol Myrzabossynov, Aigerim Zhunussova, Odiljon Dadabayev

**Affiliations:** 1 Department of Sports Business and Tourism, Kazakh National University of Sports, Astana, Kazakhstan; 2 Department of Public Administration and Project Management, Astana IT University, Astana, Kazakhstan; 3 The Ministry of Tourism and Sports of the Republic of Kazakhstan, Astana, Kazakhstan; 4 Department of Orthopedic and Pediatric Dentistry, Astana Medical University, Astana, Kazakhstan; 5 Scientific Research Institute of Physical Culture, Uzbek State University of Physical Education and Sport, Tashkent, Uzbekistan

**Keywords:** active longevity, digital skills, digitalisation, European Union, older age, physical activity

## Abstract

The digital transformation of society creates new opportunities for the involvement of older population in physical fitness and sports activities, however, the nature and the power of the impact exerted by various aspects of digitalisation on the physical activity level remain understudied. The goal of the study is to identify digital factors and to assess their impact on the physical activity of older population. The study was conducted using a sample of 27 European Union countries for the period 2015–2024 with the use of physical activity indicators of population aged 60–74 years, the level of internet usage by this age group, digital skills of population, the e-Governance development index, and gross domestic product per capita. Correlation analysis was used to identify relationship between variables under study. Statistical methods were selected taking account of the results of data distribution normality testing with the use of the Shapiro–Wilk criterion. The Granger causality test was used to measure the direction of identified relationship. Estimates were made using the Stata software. The findings of the study confirmed the existence of statistically significant positive correlation between digitalisation indicators and the level of physical activity of older population. The strongest relationship was discovered between the digital skills of population and the level of physical activity of people aged 60–74 years (*r* = 0.781; *p* < 0.01). A significant impact was also demonstrated by the internet usage by the population of older age groups (*r* = 0.712; *p* < 0.01) and the e-Governance development index (*r* = 0.637; *p* < 0.01). The Granger causality test’s results showed that digital skills precede changes in the level of physical activity in 18 European Union countries, while the internet usage in 16 countries, and the e-Governance development in 13 countries. The findings suggest that the digitalisation can be regarded as one of factors for the development of physical activity of older population. The practical significance of the study consists in the opportunity to use the findings in the development of active longevity programs, digital inclusion of older citizens, and digital transformation projects in the field of physical fitness and sports.

## 1 Introduction

Within the context of the everyday world, the problem of physical inactivity among older people grows into a global challenge for public healthcare. According to the World Health Organisation data, physical inactivity occupies the fourth place among global mortality risk factors, causing about 3.2 million deaths every year. In this regard, only 43.5% of older people in the world comply with recommendations for everyday physical activity (1).

Along with this problem, there evolves a rapid digitalisation of all spheres of life, which carries both new opportunities and serious challenges for the older generation. Rapid aging of the population—according to forecasts, by 2050 the number of people aged 60 years and above will reach 2.1 billion persons (2)—makes researchers and politicians search for effective tools for the involvement of older people in regular physical activity, and digital technologies can be one of such tools.

The use of digitalisation as a tool for promoting mass sport among older citizens becomes particularly topical in the European Union (EU) member states. These territories are characterized by a high share of older adults in the total number of population, which fact makes the authorities of European countries elaborate and implement various tools and measures for the involvement of this category of citizens in mass sport.

The EU member countries form a united area, on the whole territory of which a common digital transformation strategy and trends of active longevity development policy apply. In this context, these states show considerable differences in the digital maturity level. The Eurostat data indicate that leading positions in terms of the share of population having basic skills in the digital sphere are occupied by Finland, the Netherlands and other Northern European countries (above 80%), whereas in such states as Eastern European countries of the EU this index remains below the average values (3).

One of priority programs being implemented in the EU area is the Digital Decade project, which includes specific target benchmarks in the field of expansion of basic digital skills among the population and implementation of modern-day e-services (4). This fact makes the EU states the most relevant object for the assessment of the impact of digitalisation on the social engagement of older people in the community.

Currently, the question of ‘to what extent the general level of digitalisation of the country and the government policy in the field of digital inclusion contribute to the formation of preconditions, which promote the mass physical activity among older people’ is inadequately treated in the research literature. The quantitative study of this correlation will make it possible to determine the advisability of the digital capacity development as a measure promoting the involvement of older citizens in mass sports. For the purposes of efforts made by the states to build an inclusive digital society, this study takes on particular importance.

## 2 Literature review

In the last 10 years, many research papers were published that substantiate a high effectiveness of digitalisation as a tool facilitating the involvement of older people into regular physical activities.

Alley et al. (5) performed a comprehensive meta-analysis consisting of 22 systematic reviews on the basis of 185 primary research papers, in which 28,198 respondents took part. According to the findings, digital interventions represented by various services, activity trackers and apps have a statistically significant effect in the context of involvement of older population in regular physical activities regardless of the popularity and the type of digital solutions. At the same time, the authors point out the fact that only 14% of materials from the total number of reviews suggest a long-term nature of the above-mentioned effect, which indicates a deficiency of the body of evidence.

Similar conclusions were drawn by French et al. (6), who point out a significant effect from the use of various mobile services and apps to support the physical activity of older people.

Zheng et al. (7) performed an extensive scoping review of digital solutions and determined that with reference to the existence of a significant amount of positive feedback concerning the operation of those services, the users are still faced with such barriers in the implementation of technologies as potential safety concerns, inadequate base of knowledge in the digital sphere, and a lack of subjective assessment of compliance with requests of end users.

In their paper, Di Pumpo et al. (8) claim the lack of systematic data regarding the digital interventions used and the need to introduce a standardization of methods for the assessment of the effectiveness of proposed solutions.

The research literature actively studies not only empirical evidence of physical activity of older people but also such aspects as the result of the use of digital technologies in terms of a person’s inner state and his or her place in society. Hu et al. (9) demonstrated in their systematic review and meta-analysis of 15 randomized controlled studies that digital interventions significantly improve quality of life and mental health in older adults with chronic diseases.

Singh et al. (10) confirmed that physical activity interventions, including those implemented with the use of digital technologies, are effective for improving symptoms of depression. In their European review, Zangger et al. (11) analysed the benefits and harms of digital interventions promoting physical activity and arrived at a conclusion that advantages outweigh potential risks provided that the age-specific features of users are taken into account.

Digital literacy of older adults is critical for the effectiveness of any digital interventions, including physical activity programs. Oh et al. (12) evaluated instruments for the measurement of digital literacy in older people by analysing 27 research papers from the USA, Germany, China, Italy, Sweden, Canada, Iran, and Bangladesh.

The most frequently used instrument was the eHealth Literacy Scale (eHEALS) as it was applied in 59% of research papers specifically. The authors also showed that most of instruments cover only one or two elements of five digital literacy competencies under the European Digital Competence Framework (DigComp), whereas the Mobile Device Proficiency Questionnaire (MDPQ) covers all five elements, including “digital content creation” and “safety.” This paper points out a need to use those particular assessment instruments, which are as much as possible adapted for older adults.

Wang and Luan (13) mentioned that despite continuously growing interest in this subject, the baseline methodological positions, which set out key principles, lack consistency and integrity. As a result of their exploration of longitudinal data from the China Family Panel Studies (CFPS, 2016–2022), Yang et al. (14) obtained empirical evidence of positive impact of high digital literacy on the mental and physical health of older adults with the facilitation of social support networks and the introduction to a healthier system of living habits.

Kim et al. (15) used big data of the National Survey of Older Koreans in their study, which exploration led to the conclusion that the higher digital literacy is associated with greater life and health satisfaction.

Studies based on DigComp are of special significance for European countries. The study of Vercruyssen et al. (16) included a poll conducted among 26 instructors from Belgium teaching the basics of digital literacy for older people, who demonstrated that even elementary manipulations with digital services appear to be difficult for representatives of this age group without relevant experience. The study puts forward arguments indicating the existence of over-demanding expectations for new users of digital services, who do not understand the computer jargon and are not familiar with software at the initial stages. This issue was a target of research conducted by Jena and Paltasingh (17), who organized data on the level of digital literacy of older population in India. The authors confirmed that the nature and the degree of impact of barriers are considerably contingent on economic, social and cultural factors.

The research literature defines barriers that hinder the promotion of digital technologies and, consequently, physical activity control services among older adults. Wilson et al. (18) determined the most prevalent barriers that restrict the use of e-health services by older adults, such as narrowness of knowledge in this field, inadequacy of functionality, and lack of comprehensive information about the benefits.

Khan et al. (19) identified in their work barriers hindering the improvement of digital literacy for older adults:

- barriers for groups of people with special needs;
- support barriers;
- barriers of simplicity and usability of information and knowledge;
- lack or insufficiency of key resources;
- perception barriers.

These barriers in the aggregate exert a common adverse effect on digital engagement in the involvement of older adults in mass sports. The ways to facilitate successful overcoming of existing barriers include the intensive participation of users of digital solutions in building their design, improvement of the ability to solve specific problems independently, provision of information about methods to prevent personal data breaches, and active implementation of e-health systems.

In their review dedicated to the examination of the impact of barriers on healthcare digitalisation processes, Birati and Tzemah-Shahar (20) used the Item Response Theory (IRT) and arrived at the conclusion that the resistance should be regarded as a complex process comprising a whole list of different barriers ranging from identity-based to psychological ones. We should take note of one of the most important aspects that reinforces the avoidance behavior—the so-called feedback loops, which arise as a result of increase in technology anxiety and low self-efficacy.

At present, the most effective tools to overcome any possible barriers are programs aimed at increasing the digital literacy of underserved older adults. The study of Miller et al. (21) provided a full description of similar projects, for the implementation of which students were involved as part of community-engaged learning, and pointed out to their high efficiency in digital literacy training of older adults.

The study of Jung et al. (22) discussed digital literacy programs organized in a US public library, analysing their sustainability and indicating the need to introduce tailored inclusive courses for people of the concerned age category. Lockhart and Hunter (23) identified a strong need to improve the digital skills of older residents of small settlements in New Zealand and determined that the population can be involved in digital learning through the use of encouraging health-related instruments.

The issue of application of wearables with the aim of analysing and promoting physical activity is a separate object of study. Nagappan et al. (24) conducted an extensive poll involving 23,974 US citizens (2020–2022) and found that only 44.5% of respondents use wearables in their everyday life. In this context, the largest part of users is accounted for active young people, inhabitants of large cities and people with higher incomes. Potential usage of wearables among older adults showed a figure of 5.5 times lower than that in the group aged 18–24 years.

This data indicate a significant digital gap, which must be bridged for the full use of the potential of wearables in population-based physical activity programs.

In their systematic review of solutions based on wearable-enabled remote health monitoring, Ribeiro et al. (25) confirmed their effectiveness but also noted problems of availability for older users. Salama and Gadbois (26) considered the interpretation of wearable data (heart rate variability, sleep, stress) in sports practice thereby creating a methodological foundation for the use of such devices in mass sport programs for older adults. Adepoju et al. (27) and Zhou et al. (28) focused on the problems of introducing wearables and smart technologies in chronic disease management and the everyday lives of older adults highlighting the need to take into account age-specific cognitive and motor limitations when designing interfaces.

Memmer et al. (29) presented the SMART-AGE protocol—a randomized controlled trial examining the effect of a tablet-based app aimed at increasing social participation, physical fitness and health awareness among older adults aged 67 years and above in Germany. The trial uses three apps covering the key domains—social participation, physical fitness, and health awareness—as well as a feedback app.

In the course of their work, Böttinger et al. (30) examined data on the use of tablet based Keep On Keep Up digital solution by German residents, which was created with a view to motivating older adults for cognitive and physical activity. Such digital interventions are developed as comprehensive multi-domain programs that are more efficient and adapted than targeted interventions.

The analysis conducted allows us to identify three interrelated levels of this influence, which together form a conceptual model.

The first level—the individual-competency level—is based on the findings of studies on digital literacy among older adults (12–14). It is precisely the level of digital proficiency that serves as the fundamental resource that transforms the theoretical accessibility of technologies into the actual use of these technologies for health promotion. According to the works of Vercruyssen et al. (16) and Jena and Paltasingh (17), without developed competencies, even intuitively understandable interfaces remain inaccessible, and the motivational incentives provided by the apps fail to reach their intended audience. Thus, digital skills are a fundamental prerequisite for digital services that support physical activity.

The second level—communications and infrastructure—relates to the expansion of internet access among the older generation. As shown by research by Wilson et al. (18) and Khan et al. (19), even with a high level of digital skills, the lack of a stable connection or limited network coverage negates the potential benefits of any online programs. At the same time, the Internet serves two functions: on the one hand, it provides access to specialized platforms (video workouts, group classes, fitness trackers, etc.); on the other, it creates a channel for social support, which, according to Kim et al. (15) and Hu et al. (9), links digital engagement to psychological and emotional well-being, thereby fostering motivation for regular physical activity.

The third level—the institutional-political level—is reflected in the development of e-government. Studies of national strategies for active aging (3, 4, 31) demonstrate that the development of digital government services creates the conditions for encouraging older adults to adopt health-promoting behaviors (portals for registering for sports events, electronic health records, integration with social security systems, etc.). Such an institutional environment reduces transaction costs for older users, and the transparency of information fosters trust in digital services. This is particularly important for countries with a low level of digital literacy (20).

Thus, existing concepts can be integrated into a single three-level model for encouraging older adults to engage in physical activity: institutional support creates a favorable environment, the concept of internet accessibility provides channels and connections, but the level of individual users’ skills remains the decisive factor.

Despite an extensive scientific background, some macroeconomic issues relating to the development of government policy directions in the area of active longevity and mass sports remain underexplored so far. Firstly, most part of the studies (5, 6, 8) are dedicated to the analysis of effectiveness of certain digital services, apps and wearables by means of allocating participants to groups in a random manner or according to a common event. The outcome of these works was an internal validity report. However, in the real world, this information does not provide a complete picture about the extent of impact made by the level of digitalisation, which, in general terms, includes such aspects as the popularity of smartphones, the extent of broadband internet connection coverage, and the amount of investments in the development of digital infrastructure, on the promotion of mass sport among older adults.

Secondly, even though the studies dedicated to digital literacy (12, 14, 16) identify individual predictors and barriers, they actually do not consider them at an aggregate level. It remains unclear whether country-based indicators of digital literacy of older adults are associated with their physical activity level.

Thus, the existing studies, being predominantly clinically-oriented or qualitative in nature, do not provide a systemic, quantified answer to the question about the extent to which digitalisation serves as a significant instrument for the development of physical activity among older adults.

The goal of the study is to validate the role of digitalisation as a significant instrument for the development of physical activity among the older population.

## 3 Methods of study

This paper considers digitalisation as a potential factor and an effective instrument to increase the involvement of population in physical fitness and sports activities. The empirical background includes data of 27 EU member states for the period 2015–2024.

The EU states use Eurostat uniform methods for collecting and publishing data, thus providing for the comparability of indicators of digital skills, internet usage, and demographic profiles of population. The World Health Organization report “Health-enhancing physical activity in the European Union” (2024) includes up-to-date data on the popularity of physical activity among various age groups.

The choice of the time span is conditioned by the need to ensure the comparability of international statistics.

Despite the availability of individual digital indicators for 2025, data on the physical activity of older population for most of the EU countries were available only up to 2024 at the time of the study. The latest UN E-Government Survey was also conducted in 2024 (32), which determined the upper threshold of the reviewed period.

The indicator of physical activity of population aged 60–74 years (SPORT60_74) was used as a response variable. This indicator reflects a share of persons of the respective age group who comply with the World Health Organization guidelines of at least 150 min of moderate-intensity physical activity per week or an equivalent amount of intensive physical activity. The choice of this variable is conditioned by the fact that the physical activity is one of key indicators of well-aging and is widely used in international comparative studies (33).

Indicators characterizing various aspects of the digital maturity of society were chosen as explicative variables.

The first independent variable—I_60–74_ (Internet Usage 60–74 years)—reflects a share of population aged 60–74 years, who regularly use internet. This indicator gives the possibility to assess the extent of actual involvement of older citizens in the digital environment and describes the availability of digital services for the reviewed age group.

The second variable—D (Basic or Above Basic Digital Skills)—represents a share of population possessing basic or higher-level digital skills. The indicator is calculated by Eurostat on the basis of a set of competencies, including information search, communication, digital content creation, problem-solving, and safety support in the digital environment. The indicator is considered as a proxy for the digital literacy of population (4).

The third variable—E (E-Government Development Index)—describes the level of e-government development. The index is calculated by the United Nations and combines indicators of online services, telecommunication infrastructure, and human capital. The above-mentioned indicator can be used to perform an analysis of the institutional framework of digitalisation.

GDP (Gross Domestic Product) serves as a monitoring element reflecting the size of the national economy and the amount of new value created within it. This variable is included in the analysis in view of the fact that the level of economic development is interpreted in the research literature as one of key drivers for digitalisation and popularity of mass sports amongst the population. The characteristics of the above-mentioned variables are shown in Table 1.

**Table 1:** Variables for study.

Variables	Content	Unit of measurement	Data source
S_60–74_	Level of physical activity of people (60–74 years)	% of population	World Health Organization
I_60–74_	Share of older internet users (60–74 years)	% of population	Eurostat
D	People with basic skills and above	% of population	Eurostat
E	e-government development index	index (0–1)	United Nations
GDP	GDP per capita	USD per capita	World Bank

The correlation analysis makes it possible to assess synergies between the given indicators. At the initial stage, a calculation of descriptive statistics was made, the results of which enable us to identify cross-country inhomogeneity of the above-mentioned variables. The next stage involved data distribution normality testing. Spearman or Pearson coefficients were used to determine the results of testing.

The assessment of long-term correlations in longitudinal data was made by means of sampling, which included 270 observations. The use of a long-term body was due to the fact that it combines both the time series и spatial data analysis of various countries.

The Granger causality test was additionally used to assess the direction of identified statistical relations between the digitalisation and physical activity of population of older age groups.

Data processing was carried out with the use of IBM SPSS Statistics and Stata software packages. The statistical significance level for all calculations was assumed to be equal to 5% (*p* < 0.05), which corresponds to the common practice of empirical studies in the fields of economics, public administration, and social policy.

## 4 Findings of the study

Recently, the EU countries have been pursuing an all-European policy of active longevity as well as expansion of digital services in the areas of healthcare, sports and social care. Over the last years, in the EU there has been noted significant progress in the implementation of national physical activity development programs, while the level of introduction of the respective policies has increased from 64.7 to 81.8% (34).

To identify general trends, an initial behavior analysis of indicators under study on an EU-27 average basis was conducted (Figure 1).

**Figure 1:**
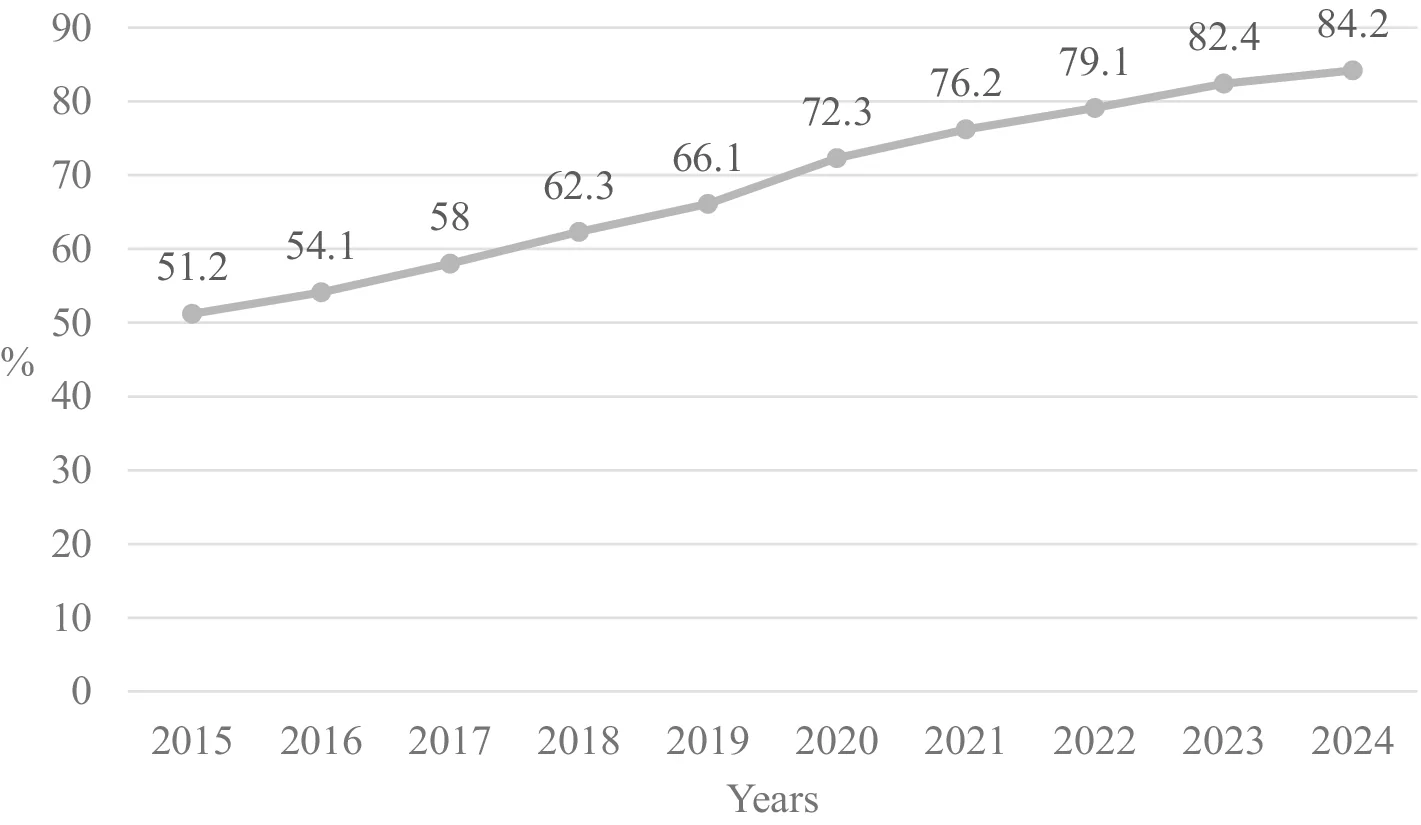
Trends of internet usage by people aged 60–74 years in the EU countries in 2015–2024, %.

For the researched period, the share of internet users among the population aged 60–74 years increased by 33%. The most intensive growth occurred after 2020, which is due to the acceleration of the digital transformation of public services, healthcare, and communication platforms. Differences between the states persisted; however, the general upward trend was typical for almost all EU countries. In North European countries, the level of internet usage among the older population approached the full coverage, while a notable digital gap persisted in a number of East European countries.

The analysis of average values of physical activity indicators of population aged 60–74 years gives evidence of the presence of a sustainable upward trend in the European Union countries over the whole period (Figure 2).

**Figure 2:**
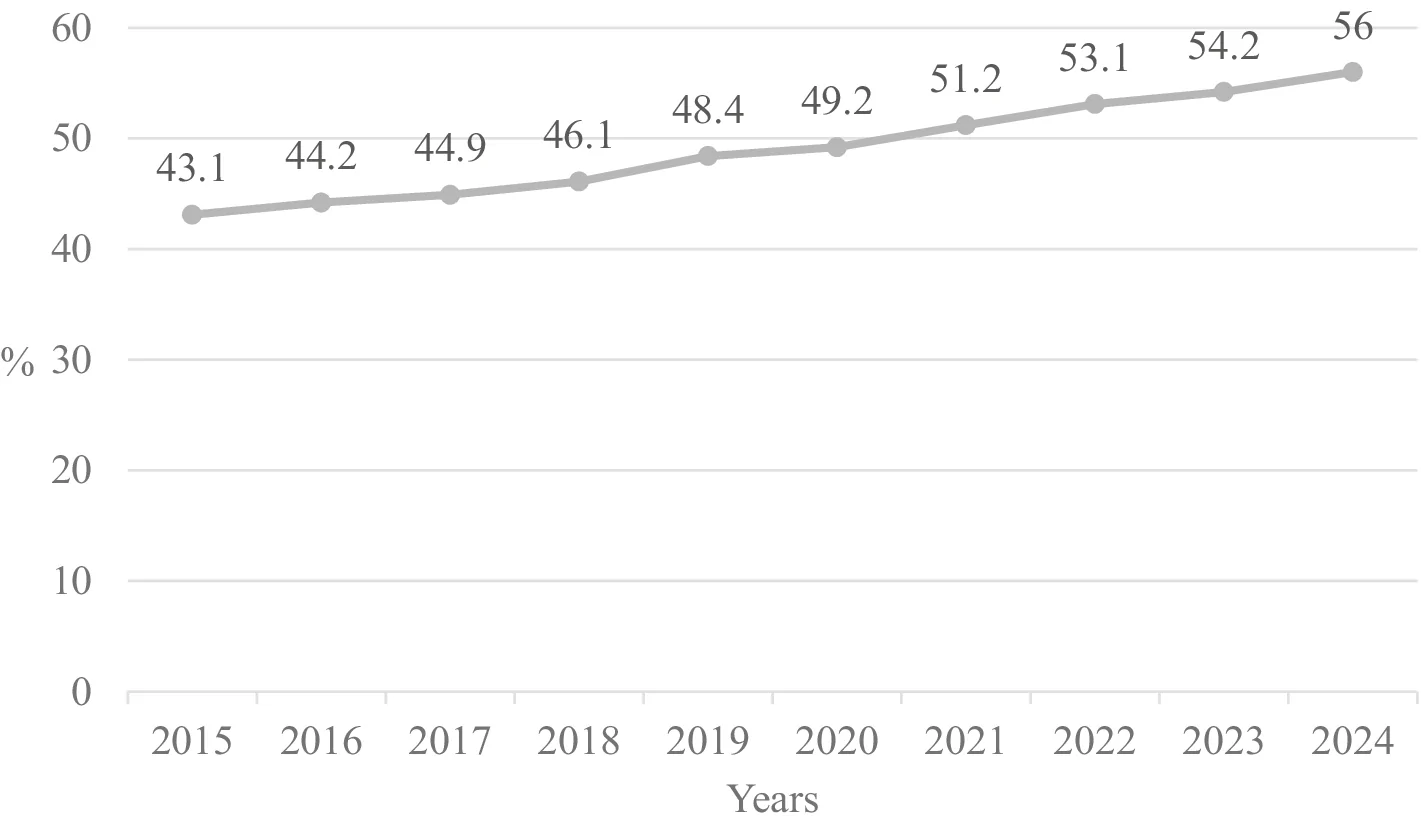
Behavior pattern of the physical activity of population aged 60–74 years in the EU countries in 2015–2024, %.

The data obtained indicate a gradual increase in the involvement of older people in physical fitness and mass sport. Despite the upward trend, the level of physical activity of older people remains essentially low. According to OECD estimates, on average across the EU, only about one-fifth of persons aged above 65 years fulfil the recommended physical activity standards (35).

The positive trend shown in Figure 2, combined with the persistently low absolute level of physical activity among older adults, indicates that behavioral patterns in this age group are resistant to change. Even with a steady upward trend, surpassing the 25% threshold of those engaged in regular physical activity will require not an extensive expansion but a qualitative change in the motivational structure. The observed increase indicates the beginning of systemic shifts rather than the completion of the formation of health-promoting behavior. Consequently, the task is not to consolidate progress but to identify levers for accelerating the transition from sporadic to widespread participation of older adults in physical activity.

To assess the cross-country inhomogeneity, the calculation of average values of indicators for the whole reviewed period was made (Table 2).

**Table 2:** Leading and lagging countries in terms of the average level of digital engagement of population aged 60–74 years in 2015–2024, %.

Leading countries	Value
Netherlands	92.1
Denmark	91.3
Finland	90.8
Sweden	90.4
Luxembourg	89.7

Lagging countries	Value
Romania	45.7
Bulgaria	49.3
Greece	58.2
Croatia	61.5
Hungary	63.8

This distribution of countries reveals a significant institutional gap that is reflected in the structure of everyday practices. For example, the fact that the Netherlands’ rate (92.1%) is several times higher than Romania’s (45.7%) cannot be explained solely by economic disparities. The reasons lie in differences in social policy priorities, established traditions of intergenerational continuity in this behavior, and public trust in government services. A vicious cycle is at play here: low levels of access limit opportunities for learning, while a lack of practical skills reinforces the older generation’s passive resistance to new technologies.

The results demonstrate the presence of a steady digital inequality between the countries of the Northern and Eastern Europe. A similar gap is also observed with regard to physical activity (Table 3).

**Table 3:** Leading and lagging countries in terms of the average level of physical activity of population aged 60–74 years in 2015–2024, %.

Leading countries	Value
Sweden	68.5
Finland	67.8
Netherlands	66.9
Denmark	65.7
Ireland	61.4

Lagging countries	Value
Romania	29.8
Bulgaria	32.4
Greece	37.1
Croatia	39.6
Hungary	41.8

The overlap between the top performers and the bottom performers in the rankings for physical activity and digital engagement may indicate the possible existence of a common macro-factor. This could be the quality of human capital and/or the degree of social cohesion, which together determine a similar level of trust and institutional support. However, this does not imply a clear-cut direct causal relationship. It is possible that physical activity and digitalization reinforce each other in this age group. Overall, an analysis of trends in Northern and Southeastern Europe suggests the existence of stable regional clusters, which calls for a differentiated approach when developing and promoting active aging programs.

It is necessary to examine the relationship between a society’s level of digital maturity and the popularity of a physically active lifestyle among older adults. To test this hypothesis, we will analyze the correlation (Table 4).

**Table 4:** Descriptive statistics of indicators under study (EU-27, 2015–2024).

Indicator	Average	Minimum	Maximum	Standard deviation
S_60–74_	52.7	24.3	74.8	11.9
I_60–74_	78.4	29.1	96.8	15.7
D	57.6	18.7	84.5	13.1
E	0.841	0.621	0.982	0.091
GDP	34,782	8,913	94,208	17,436

It should be noted that the range of values for the physical activity indicator (standard deviation of 11.9 percentage points) is smaller compared to digital skills (13.1 percentage points) and Internet use (15.7 percentage points). Consequently, aggregated indicators of digitalization vary over a wider range, while behavior in the form of physical activity is more smoothed out (an averaged response to diverse stimuli). This indirectly suggests that the impact of digitalization on physical activity is mediated by other intermediate variables (health status, climatic conditions, availability of sports infrastructure, etc.), which reduce the significance of digital disparities at the level of final behavior. When building predictive models, it is important to consider not only the direct effects of digital indicators but also their interaction with these influencing factors. This is because in countries with low health standards, even high digital literacy may not lead to a significant increase in physical activity due to the health limitations of the older adults themselves.

The greatest variation is observed in indicators of internet usage and the digital skills of people. This is indicative of significant differences in the level of digital transformation of the EU member states. The matrix based of the results of the correlation analysis is shown in Table 5.

**Table 5:** Correlation matrix of indicators under study.

Variables	SPORT60_74
I_60–74_	0.712***
D	0.781***
E	0.637***
GDP	0.543**

***p < 0.01; **p < 0.05.

The conducted correlation analysis suggests the presence of a statistically significant positive correlation between the presented indicators describing the level of digitalisation and the physical activity of older population.

The correlation coefficient between the popularity of internet and the level of physical activity of older adults (*r* = 0.712) gives evidence of the digital accessibility transition to the group of key drivers for the promotion of mass sport among the older population.

The strongest correlation is observed between the level of digital literacy and the physical activity of older people (*r* = 0.781). The obtained result suggests that the ability to make efficient use of digital technologies is an important condition for engaging the older population in modern sport activities. At the same time, the degree of correlation varies across different EU countries (Table 6).

**Table 6:** Distribution of the EU countries by the strength of the identified relation between digital skills and physical activity of population aged 60–74 years.

Characteristics of relationship	Number of countries
Very high (*r* > 0.80)	8 (Netherlands, Denmark, Sweden, Finland, Ireland, Luxembourg, Estonia, Austria)
High (0.70 < *r* < 0.80)	9 (Germany, France, Belgium, Slovenia, the Czech Republic, Lithuania, Latvia, Malta, Portugal)
Moderate (0.50 *r* < 0.70)	7 (Spain, Italy, Poland, Slovakia, Croatia, Cyprus, Hungary)
Weak (*r* < 0.50)	3 (Greece, Bulgaria, Romania)

The positive correlation was registered in most EU states. Especially stable dependence was observed in the Netherlands, Finland, Sweden, Denmark, Ireland, and Estonia. At the same time, in Bulgaria, Romania, and Greece the statistical significance of the identified relationship appeared to be considerably lower, which may be attributable to the influence of additional institutional and socio-economic factors. The weakening of the statistical correlation across these three countries can be interpreted separately based on “hedonic displacement.” This effect means that digitalization, when implemented without taking into account age-related motor and cognitive limitations, may not only fail to stimulate physical activity but may also reinforce a sedentary lifestyle. The latter is linked to the fact that screen time increases while traditional outdoor activities (gardening, walking, going to the store, etc.) decrease.

In the absence of adapted interfaces and accessible (easy-to-understand) usage scenarios for specialized sports apps, older users in such countries may limit themselves to passive content consumption (news, social media, videos, etc.). It is also possible that, in this case, the low correlation is an artifact of the small absolute values of the indicators. That is, when physical activity levels are below 30%, external influences—including digital ones—may be drowned out by stronger determinants (such as low life expectancy in good health, chronic diseases, etc.). Consequently, in developing countries, the primary goal should not be digitalization as an end in itself, but rather improving overall health, access to basic healthcare services, and general literacy regarding healthy lifestyles. Against this backdrop, digital tools will be able to realize their full potential.

To determine the causal direction of identified relationships, the Granger causality test was used (Table 7).

**Table 7:** Results of the Granger causality test between the indicators of digitalisation and physical activity of people aged 60–74 years in the EU countries (2015–2024).

Influence	Number of countries where the hypothesis was confirmed (*р* < 0.05)
D → S_60–74_	18
I_60–74_ → S_60–74_	16
E → S_60–74_	13
S_60–74_ → D	4
S_60–74_ → I_60–74_	3
S_60–74_ → E	2

The results of the Granger causality test give evidence of the predominance of unidirectional influence of digitalisation on the physical activity of older population. The strongest causal relationships were identified for the digital skills indicator. In 18 EU countries, the changes in the D indicator statistically significantly preceded changes in the level of physical activity of population aged 60–74 years.

A similar situation is observed with regard to the internet usage indicator. It is determined that in 16 states under study there has been registered a stable positive correlation between the expansion of the digital engagement of older people and the extent of their participation in mass sport. However, the engagement of older population in physical fitness and sports activities rarely has a statistically significant influence on digitalisation indicators.

The highest level of causality was observed in such countries as Luxembourg, Denmark, Estonia, Finland, the Netherlands, and Ireland. Data regarding Bulgaria, Greece, and Romania did not lead to identifying any statistically significant causality.

Thus, the digital transformation of society can be regarded as one of positive factors contributing to the expansion of the level of engagement of older people in regular physical fitness and sports activities. At the same time, the development of digital competencies leads to the active use of various solutions (mobile services and apps, online platforms and instruments for the furtherance and support of regular physical activity).

## 5 Discussion

The results of the conducted study give evidence of the presence of a stable positive correlation between the digital maturity of society and the level of engagement of people aged 60–74 years in physical fitness and sports activities. For this purpose, the most significant factor was the level of digital literacy of people, whilst the internet usage and the e-government development demonstrated somewhat less apparent but statistically significant influence. The results obtained make it possible to regard the digitalisation not only as a technological process but also as one of elements of the institutional environment facilitating the promotion of active longevity activities.

The conclusions reached partially agree with the results of study by S. Alley et al. (5), which, on the basis of meta-analysis of 22 systematic reviews and 185 primary research papers, confirmed the effectiveness of digital interventions to promote physical activity in older adults. The authors identified the positive effect of digital solutions on the number of steps, the total amount of physical activity and the level of moderate and vigorous intensity. At the same time, the study of Alley et al. (5) is focused predominantly on the assessment of the effectiveness of individual digital interventions at the level of individual behavior.

The advantage of this study lies in the fact that the analysis was performed at a macro level and covers all EU states over a 10-year period. Such an approach made it possible to analyse the degree of impact exerted by the set of digital development indicators of the European community, including the level of digital literacy of citizens, as well as the quality of e-government services. Furthermore, this study, as opposed to the meta-analysis of Alley et al. (5), was conducted by means of sampling specifically within the age of 60 to 74 years, which allows us to achieve a more meaningful comparison of the results in the category of “older adults” in accordance with the WHO approach.

Similar results were obtained in the experimental study of Baubonytė (36) for examining of the effect of digital skills training on the use of physical activity services by older women. As alleged by the author, a nine-week digital literacy training course gives rise to the development of digital competence, promotes the increase in the willingness to use various digital sports services and regularly do physical exercises.

A significant deficiency of this study is the limited nature of sampling, which includes only 63 participants, and the analysis of mainly the degree of willingness rather than actual indicators of physical activity. In this context, our study is based on panel data regarding the population of 27 EU member states over a decade, which makes it possible to trace the actual behavior of the indicator of older people’s engagement in mass sports. Consequently, the obtained results confirm the findings of Baubonytė (36) at a higher level of data aggregation and demonstrate that the effect of digital literacy is manifested not only at the individual level but also at the level of community.

Worthy of separate attention is the comparison of results with the studies dedicated to digital technologies as an instrument to support healthy aging. The survey of Xiao et al. (37) emphasizes that most part of existing digital solutions for older adults are focused on the issues of healthcare and caregiving, while the impact of digital environment on the formation of everyday behavior patterns remains understudied. The authors also note the significant fragmentarity of the existing surveys and the lack of joint understanding of factors determining the success of digital solutions for older generation.

Against this background, the advantage of this study is the integration of two research areas that are usually examined independently: studies of digital transformation and studies of physical activity of people. This approach made it possible to determine that the digital literacy serves not merely as a driver for the adaptation of older adults to digital technologies but also as one of foundations to build a rational approach to health. Consequently, this study extends the frontiers of conclusions previously drawn by the scholarly community regarding social changes in society caused by the development of digitalisation.

Another equally important area of comparison is the analysis of the effectiveness of digital services for changing behavior. For example, Wiratunga et al. (38) described in their work a voice-based FitChat chatbot developed to encourage physical activity in older adults. The authors determined that various digital interventions motivate older adults to engage in physical activity since they are perceived more favourably than text notifications.

A significant deficiency of similar works is the fact that, in terms of the practical relevance, such studies are dedicated to the analysis of specific technological developments, which makes it harder to assess their impact for the purpose of wide use at the national level. This study examines the digital environment as an entirety of interrelated institutional, competence-based, and infrastructural elements, which gives an opportunity to make up for deficiencies of similar studies. In the process of analysis, we identified a positive consistent change in the level of digital skills and physical activity, which may indicate a dependence of the effectiveness of certain instruments on the degree of digital readiness of people.

One of the key conclusions of this study is a critical difference in the level of digitalisation development across the European Union countries. The highest indicators of digitalisation and physical activity of older adults have been recorded in the northern states—Finland, Sweden, Denmark, and the Netherlands. In Bulgaria, Romania, and Greece the respective indicators are lower. This indicates that the level of digitalisation and physical activity can be interpreted as components of a scaled model of social and economic development.

This study is based on longitudinal data collected over a long-term period and the Granger causality test. This distinguishes this work from similar studies, which are limited to experimental samples or cross-sectional analysis, thereby making it possible to determine a statistical relationship and assess the vector of its formation. According to the results, in most states an increase in physical activity of older adults is associated with the preceding behavior of digitalisation indicators. In the absence of satisfactory arguments for the interpretation of the above-stated conclusion as evidence of strict causality, it suggests that the digital transformation serves as a factor to build a foundation for promotion of physical activity.

In light of the foregoing, we can make a conclusion that within the framework of similar studies, this work has numerous advantages: the use of cross-country longitudinal data of all countries of the European Union; analysis over a 10-year period; and examination of digitalisation as a complex phenomenon consisting of such elements as digital engagement, digital skills, e-governance, the application of correlation analysis and statistical dependence vector testing. This approach made it possible to get a larger-scaled vision of how digital solutions influence the involvement of older adults in mass sport and to enrich previously obtained findings of other studies examining the state policy in the field of active longevity.

However, in the presence of statistically significant conclusions, the work has some limitations to be taken into account when interpreting them.

One of main deficiencies is the use of international statistics in the study, which has certain specifics. The aggregated data across the European Union states examined in the course of the analysis made it possible to identify institutional and macroeconomic causal relationships, but limited the opportunities for examining individual behavioural processes and patterns that bring older adults to mass sport. This limitation does not allow for the conclusion that all users of digital services and apps are fully engaged in physical activities.

We should also take note of factors related to the limitation of European territories. The overwhelming majority of the EU member states have achieved a high level of institutional, social and digital development. This fact hinders the application of the findings made in this work with regard to countries with a lower level of social and economic development. It is expected that in the future this study will be supplemented with an analysis of other countries outside the European Union. The use of data on countries of the Middle East, Latin America, Eastern Europe, and Asia will make it possible to assess statistically significant findings of the study in other cultural and institutional conditions.

Further on, research papers can be based on various fixed- and random-effects models, causal analysis, dynamic panel models, and structured modelling. This will allow itemizing and making more specific the degree of impact made by digitalisation on the level of involvement of older adults in physical activity.

Nevertheless, the findings can already be used to improve existing strategies for active aging. First, the finding that digital skills have a sustained positive impact on physical activity levels—observed in most EU countries—calls for a shift in focus from simply expanding Internet access to providing intensive training in digital literacy for older users. Second, the significant role of e-government highlights the need to implement specialized digital platforms in this area (integration with electronic health records and benefit systems, one-stop shops for registering for classes at municipal organizations, etc.). Third, the identified stratification of countries (northern and southeastern clusters) underscores the importance of differentiating roadmaps. Overcoming “digital exclusion” through training centers should become a priority for lagging regions. Leading countries need to scale up personalized wearable solutions with feedback capabilities. Furthermore, as part of a unified EU policy at the federal level, it is advisable to establish cooperation between leading and lagging countries to exchange experiences on digitalization and the promotion of mass sports. Such measures will help transform digitalization into a real tool for increasing physical activity among older adults.

## 6 Conclusion

The result of this study is the identification of the strength, nature and direction of the relationship between the development of digitalisation of society and the promotion of mass sport among people aged 60 to 74 years residing in the European Union member states. On the basis of correlation analysis we established the statistically significant relationship between the extent of older adults’ engagement in physical activity and indicators of digitalisation presented in the study.

A particular relationship is observed between the level of development of people’s digital skills and the level of physical activity of people in the above-mentioned age group (*r* = 0.781; *p* < 0.01). A less strong relationship was identified between the use of internet by older adults (*r* = 0.712; *p* < 0.01) and the development index of e-governance systems (*r* = 0.637; *p* < 0.01). On the other hand, the level of economic development was characterized by a moderate positive correlation with physical activity of older adults and their engagement in mass sport (*r* = 0.543; *p* < 0.05).

The use of the Granger causality test showed that the digital skills of people precede the changes in the level of physical activity in 18 countries of the European Union. With regard to the internet usage indicator, a similar relationship was identified in 16 countries, and with regard to the e-governance development index—in 13 countries. A feedback effect of physical activity on digitalisation indicators was observed significantly less frequently. This suggests that the digital transformation of society may serve as one of the drivers for the formation of conditions to expand the participation of older adults in physical fitness and sports activities.

When comparing the impact of the examined digitalisation factors on the level of physical activity of people aged 60–74 years, it should be noted that it is the digital skills of people that matter the most. As opposed to simple internet access or the availability of a well-developed digital infrastructure, the ability to make efficient use of digital technologies has a more significant effect on the engagement of older adults in mass sport activities. Consequently, the development of digital literacy can be regarded as one of the most effective instruments for increasing the physical activity of older adults.

The results obtained can be used in the development of national active longevity programs, strategies for the digital transformation of the sports sector, and mass sport development projects.
